# COVID-19 induced economic loss and ensuring food security for vulnerable groups: Policy implications from Bangladesh

**DOI:** 10.1371/journal.pone.0240709

**Published:** 2020-10-16

**Authors:** Khondoker Abdul Mottaleb, Mohammed Mainuddin, Tetsushi Sonobe

**Affiliations:** 1 Socioeconomics Program, International Maize and Wheat Improvement Center (CIMMYT), Texcoco, Mexico; 2 Surface Water and Basin Outcomes Group, Water Security Program, CSIRO Land and Water, Black Mountain Laboratories, Canberra, ACT, Australia; 3 Asian Development Bank Institute, Chiyoda-ku, Tokyo, Japan; Kansas State University, UNITED STATES

## Abstract

At present nearly half of the world’s population is under some form of government restriction to curb the spread of COVID-19, an extremely contagious disease. In Bangladesh, in the wake of five deaths and 48 infections from COVID-19, between March 24 and May 30, 2020, the government imposed a nationwide lockdown. While this lockdown restricted the spread of COVID-19, in the absence of effective support, it can generate severe food and nutrition insecurity for daily wage-based workers. Of the 61 million employed labor force in Bangladesh, nearly 35% of them are paid on a daily basis. This study examines the food security and welfare impacts of the COVID-19 induced lockdown on daily wage workers both in the farm and nonfarm sectors in Bangladesh. Using information from more than 50,000 respondents complied with the 2016–17 Household Income and Expenditure Survey (HIES) in Bangladesh, this study estimates daily wage rates as Bangladesh Taka (BDT) 272.2 in the farm sector and BDT 361.5 in the nonfarm sector. Using the estimated daily wage earnings, this study estimates that a one-day complete lockdown generates a US$64.2 million equivalent economic loss only considering the wage loss of the daily wage workers. After estimating the daily per capita food expenditure separately for farm and nonfarm households, this study estimates a minimum compensation package for the daily wage-based farm and nonfarm households around the US $ 1 per day per household to ensure minimum food security for the daily wage-based worker households.

## 1. Introduction

By September 14, 2020, nearly 29 million people in 216 countries and territories have been sickened by the severe acute respiratory syndrome coronavirus-2 (SARS-CoV-2) or COVID-19 [1]. By that time, the COVID-19 induced death toll had reached more than 925 thousand globally [1]. The virus was first detected on December 31^st^ 2019 in Wuhan Province, China, and on January 11, 2020, China confirmed the first COVID-19 induced death followed by the first death in the United States on January 21, 2020 [2]. On January 30, 2020, the World Health Organization (WHO) declared COVID-19 a global public health emergency of international concern [3].

COVID-19 is an extremely contagious disease, spreading rapidly through human to human contact [4–6]. Until the time of writing this article, there is no effective medicine to cure or vaccine to protect from this virus. To curb the spread of this disease, national governments have imposed varying levels of movement restrictions. For example, by April 7, 2020, national governments of 32 countries and territories in Asia, 43 in Europe, 38 in the Americas and the Caribbean, and 44 in Africa imposed varying levels of movement restrictions [7]. Studies and opinions warned that the COVID-19 lockdown and restricted labor movement could generate havoc across the world [8, 9], and could cause severe global food shortages by disrupting the supply chain [10–18]. Particularly, the one-size-fits-all approach of lockdown can aggravate the plight of daily wage workers in low-income countries. Considering this, one suggestion is to expand social safety net programs in developing countries [14]. However, to our knowledge, there is no solid study based on microdata that examines the economic loss due to the COVID-19 induced lockdown and the minimum compensation required to ensure the basic nutrition of the poor households.

The present study uses information from 50,000 economically active workers in Bangladesh, collected by the Bangladesh Bureau of Statistics (BBS), to quantify the economic loss due to the COVID-19 lockdown based on the lost wage earnings of the daily wage workers in the farm and nonfarm sectors of Bangladesh. Then, applying simple econometric estimation processes, this study estimates the minimum compensation packages for the daily wage-based farm and nonfarm households of Bangladesh that ensure their minimum food security during the lockdown. The rest of the study is organized as follows. The next section describes the current poverty situation and the COVID-19 induced restricted movement and lockdown in the world and Bangladesh. Section 3 explains materials and methods, and section 4 presents major findings. Section 5 provides conclusions and policy implications.

## 2. COVID-19 can worsen the global poverty situation

To control the spread of COVID-19, at least 157 countries and territories in the world imposed varying levels of movement restrictions [7]. In Asia, 12 countries implemented localized lockdowns, 10 countries implemented national lockdowns, and the rests implemented localized or suggested national recommendations, such as maintaining social distance [7]. These COVID-19 induced lockdowns and movement restrictions can exacerbate the already worsening global poverty situation.

Despite the extraordinary success in alleviating abject hunger and extreme poverty since the 1990s [19], the share of hungry people in the globe has been increasing in recent years [20, 21]. In 1950, more than 63% of the total population of the world was extremely poor, living on less than US$ 1.90/day [19]. In 1990, globally, the extremely poor reduced to 35.9% (1,895 million), which further reduced to 20.8% (1,352 million) in 2005 [22]. However, since 2015, the absolute number of hungry people has started to increase. In 2015, out of a global population of 7.3 billion, 784 million (10.7%) were hungry, which increased to 821 million (10.9%) in 2017 [23].

It is projected that COVID-19 will further exacerbate the poverty and abject hunger in the world in a number of ways. It is argued that the overburden on the health sector causes the reallocation of resources to the health sector to save lives, which may reduce resource allocation to the agriculture and industrial sectors, thereby hampering the food production and input supply chains [16]. More importantly, COVID-19 induced lockdowns and movement restrictions have already increased unemployment and under-employment, which has severely reduced the purchasing power of consumers [10]. The International Labor Organization (ILO) estimates that the participation of the global working force in the first quarter of 2020 declined by 4.5% which is equivalent to 130 million full-time jobs [8]. In a recent report, Asian Development Bank (ADB) projected that the COVID-19 induced global loss will range between $6.1 to $9.1 trillion compared to a no-COVID-19 baseline, which is equivalent to 7.1% to 10.5% of the global GDP [24]. The International Monetary Fund (IMF), warned that due to the COVID-19 induced lockdown and resource reallocation to the health sector to save lives, the global GDP could be reduced by 4.9% in 2020 compared to the previous year [25]. The Economic Commission for Latin America and the Caribbean (ECLAC) and ILO [26] projected that due to the COVID-19 pandemic, the economy of the Latin American and Caribbean region will shrink by 5.3% and the unemployment rate will increase from 3.5% to 11.5%, equivalent to more than 11.5 million new unemployed persons. Furthermore, the poverty rate in the region will increase by 4.4% and the extreme poverty rate will increase by 2.6% [26]. Studies also warned about a possible severe food crisis, particularly in countries currently experiencing economic shocks, weather extremes, or conflicts [10, 27].

It is observed that during epidemics like HIV/AIDS and Ebola, food prices in affected countries increased significantly, with severe impacts on food security, especially for vulnerable populations including women, children, and marginal people [28]. For example, in Sierra Leone, the triennium average ending in 2013 for rice (paddy) and cassava prices were US$614.3/ton and US$303.7/ton, respectively [29]. However, when the Ebola epidemic hit the country in 2014, the price of rice (paddy) rose to US$707.3/ton (+15%) and the cassava price to US$ 521.1/ton (+72%) [29]. Increased food prices can disproportionately affect vulnerable groups, such as marginal households and women [30, 31]. In Bangladesh, for example, the food price hikes of 2007–08 pushed an additional 13 million people below the poverty line [32].

Given the COVID-19 induced chaos, if major food and cereal exporting countries restrict their exports, a severe crisis may be generated in the food and cereal import-dependent countries [28, 33]. In 2008, droughts in Australia and Argentina and rising oil prices prompted a number of major cereal exporting countries such as India to ban cereal and food exports, aggravating the food price crisis [28]. It is reported that Kazakhstan has already banned wheat flour exports and restricted exports of buckwheat, vegetables, onions, carrots, and potatoes; while Vietnam has temporarily stopped rice exports [11]. In fact, in its latest report, the United Nations warned that world trade could shrink by 15%, the global economic output could be slashed by US$ 8.5 trillion over the next two years, and the economic growth of developed countries could reduce to -5%. Consequently, the global economy is projected to shrink by 3.2% and some 34.3 million people are projected to fall below the poverty line due to COVID-19 [34]. UNU-WIDER warned that in the most extreme case, COVID-19 induced responses could reduce 20% of the income or consumption expenditure at the global level, and poverty levels could increase by an additional 420–580 million people compared to 2018 levels [35]. COVID-19 could create a severe setback to attaining the zero hunger goal of the United Nations by 2030 [23].

In 2019, a total of 135 million people in the world were severely food insecure [10]. Food insecurity triggered by conflicts affected 77 million people in 22 countries; economic shock-related food insecurity affected 22 million people in eight countries, and weather extremes caused food insecurity for 34 million people in 25 countries [10]. The World Food Program (WFP) cautioned that in the absence of swift and effective actions, the number of severely food insecure people could reach 265 million in 2020 [27]. In addition, at present, nearly 2 billion people in the world are moderate to severely food insecure [36]. These food vulnerable people are mostly concentrated in Africa, West Asia, and Latin America [36]. Although COVID-19 has been affecting high-and low-income countries, the aftermath of COVID-19 could have severe negative impacts on low-income developing countries.

**Fig 1:**
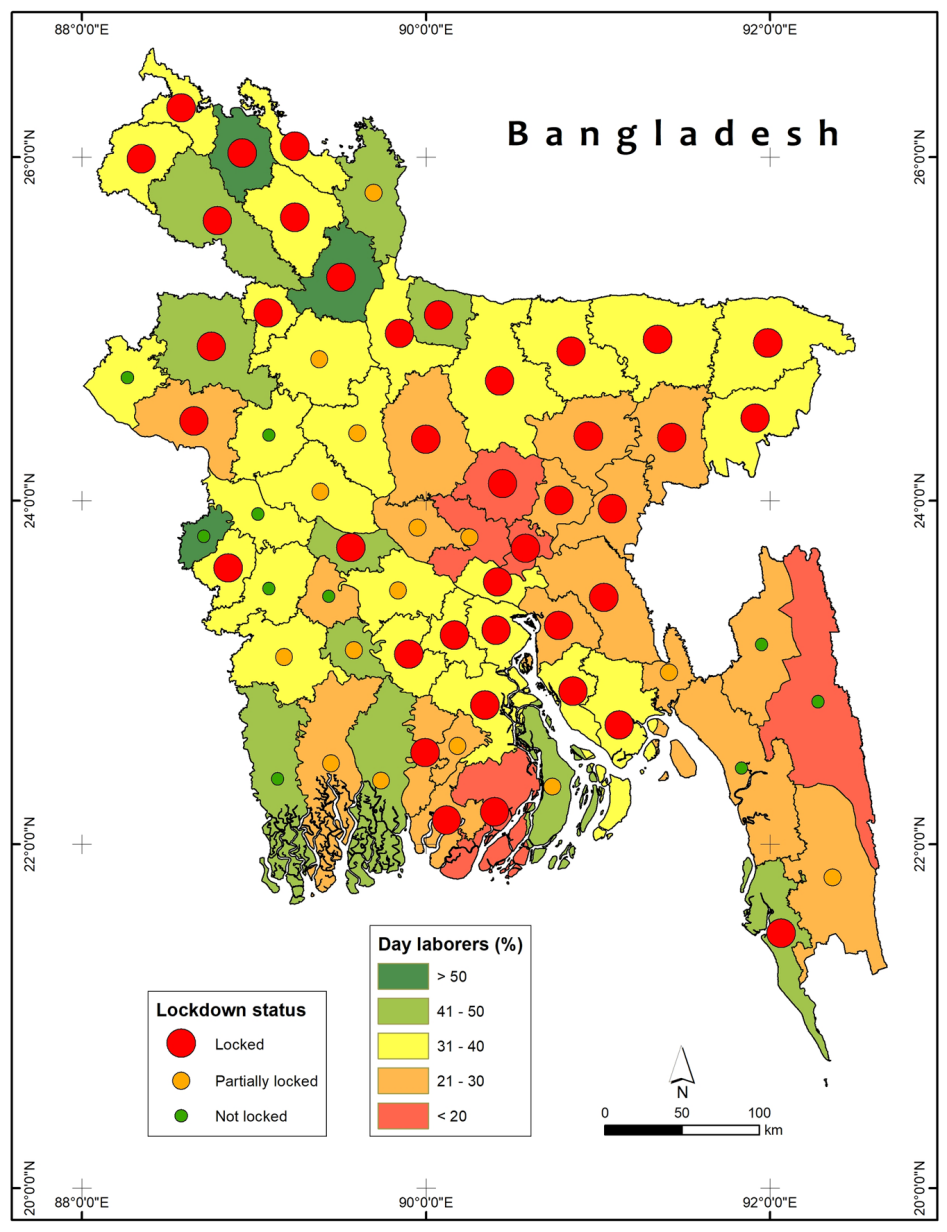
Lockdown status of the districts in Bangladesh and the share of the daily wage-based workers by districts. Sources: Authors’ based on The Business Standard; HIES 2016–17 data of BBS.

In Bangladesh, on March 23, 2020, in the wake of three deaths and 25 infections of COVID-19 [37], the Bangladesh government imposed a nationwide holiday and lockdown initially from March 26 to April 4, 2020 [38], and later extending the general holiday to April 11, and then to April 30, 2020 [39]. Finally, the government extended the general holiday and airport and road lockdown until May 30, 2020 [39]. Until April 17, 2020, out of 64 districts of Bangladesh, 38 districts were completely under lockdown and 17 were under partial lockdown [40] (Fig 1). As of September 14, 2020, Bangladesh had a total of 341,056 confirmed COVID-19 cases, with 245,594 recovered and 4,802 deaths [37].

Since 2000, Bangladesh has continued to achieve stunning economic progress. After independence in 1971, the per capita GDP of Bangladesh was $133.6, which had increased to $418.1 in 2000, and $1688 in 2018 [41]. Due to the enormous population pressure, the average farm size in Bangladesh is as small as 0.68 ha [42]. Despite the fact, Bangladesh became the fourth-largest paddy-rice-producing country (49.5 MMT) in the world after China (214 MMT), India (169 MMT), and Indonesia (81.4 MMT) [43]. Today, Bangladesh is self-sufficient in rice, the major staple of the country. The 1971–73 triennium average daily per capita dietary energy intake was 1963 kcal, which has increased to 2574 kcal/daily in 2015–17 [44]. In 2019, Bangladesh ranked 83^rd^ out of 113 countries on the Global Food Security Index [45], whereas it ranked 102^nd^ out of 119 countries in 2006 [46].

The COVID-19 induced turmoil, however, could substantially undermine the economic achievement of Bangladesh by affecting trade and the garment industry of Bangladesh. Bangladesh is the second largest garment exporting country in the world after China, where, in more than 4,000 garment factories, more than 4.4 million workers are employed, the majority of whom are female [47]. More than 80% of the total export earnings of the country solely come from the garment industry [48]. This industry is thus dependent on foreign export demand, especially from developed economies and also on imported materials from abroad and particularly from China. In 2015, the total merchandise trade value (export + import) of commodities and goods (excluding services) of Bangladesh was US $79.8 billion, of which total trade value with China was $11.1 billion [49], which was nearly 14% of the total commodity trade value of Bangladesh. In March 2020, Organization for Economic Co-operation and Development (OECD) projected that in 2020 global GDP will drop by 2.4% compared to 2019, and China’s GDP growth rate would be below 5% [50]. Thus, negative impacts of COVID-19 in China and in other high-income economies could be transmitted to Bangladesh through the commodity trade channel. Alarmingly, it is already reported that the unemployment rates in China and the United States have increased to unprecedentedly high levels [51]. In Bangladesh, reportedly, until August 2020, export order worth of US $ 3.18 billion has been cancelled, which affected the employment of 2.28 million garment workers [52].

The nationwide lockdown and movement restriction can also severely undermine the economic achievement of Bangladesh by directly curbing the wage earning opportunity of the economically employed labor force. In 2016–17, the economically active employed labor force (15 years and above) of the country was 60.8 million, of which 85.1% were informal workers (Table 1). The share of informal workers is the highest in the farm sector, in which out of total 24.7 million employed workers, 95.4% of them are informally employed [53]. The International Labor Organization (ILO) stressed that the income opportunity of the informal workers and casual labor in both farm and nonfarm sectors are mostly affected by the COVID-19 induced contraction of employment and the restricted movement measures [8]. The severe labor shortages problem in harvesting *boro* rice (dry-season irrigate rice crop) in 2020 has not only spoiled farmers’ harvest, but also curbed income opportunity of the casual labor, who earn a major portion of their yearly from harvesting *boro* rice. Consequently, among the different groups, daily wage laborers are the most food vulnerable group during this COVID-19 induced lockdown in Bangladesh [38].

**Table 1:** Employment dynamics in Bangladesh during 2002–2017.

Sector	2002/03	2005/06	2009–10	2013	2016/17
Economically active population 15+ (million)	46.3	49.5	56.7	60.7	63.5
Employed population, (million)	44.3	47.4	54.1	58.1	60.8
No. of workers in farm sector (%)	22.9 (51.7)	22.8 (48.1)	25.6 (47.3)	26.2 (45.1)	24.7 (40.6)
No. of workers in nonfarm sector (%)	21.4 (48.3)	24.6 (51.9)	28.5 (52.7)	31.9 (54.9)	36.1 (59.4)

Source: BBS [53, 64].

In 2000, 48.9% of the total population of 127.7 million, were classified as poor, which reduced to 31.5% in 2010 [41]. However, in 2016, out of 158 million people, 24.3% were classified as poor [41]. In 2015, children under 5 years of age, every 2 children in 5, were stunned, and 14% were wasted [54]. A recent report warns that if lockdown continues for a long time, the current poverty rate and undernutrition could deteriorate significantly [55]. For example, it is stressed that in Bangladesh, at least 13 million extra people have fallen below the poverty line due to COVID-19 [56]. A recent survey asserts that 75% of the sampled respondents reported not having enough food, while 91% reported of not having enough money to purchase food [38].

What is missing in the existing literature is an estimate of the magnitude of the economic loss associated directly with the lockdown measure taken by the government to curb the spread of the coronavirus, as opposed to the estimate of overall negative impacts. Using Bangladesh as a case, this study quantifies the daily economic loss due to wage earning forgone of daily wage-based workers both in farm and nonfarm sectors. As to food security, it has already been suggested to expand the public support system [14]. The present study, in addition, suggests that the minimum compensation package should be provided to ensure the minimum food security of the daily wage-based workers.

## 3. Material and methods

### 3.1 Data

This study relies on Bangladesh’s 2016–17 Household Income and Expenditure Survey (HIES) data collected by the Bangladesh Bureau of Statistics (BBS). Since 1971, BBS has conducted 15 rounds of surveys, with the HIES 2016–17 survey being the 16^th^ series. The monetary poverty of the country is measured mainly based on the HIES, which is a nationally representative comprehensive survey [57]. To enhance data precision by improving the data collection process, the World Bank provides technical support. BBS uses a two-stage stratified random sampling process. In the first stage, primary sampling units (PSUs), consisting of specific geographic areas, are selected. In the second stage, from each PSU, 20 households are randomly selected for a detailed interview. The 2016–17 dataset comprises 2,304 PSUs covering all 64 districts of Bangladesh and 46,080 households.

The 2016–17 HIES survey is a multipurpose survey, with nine sections detailing information on household level demographics, food consumption, farm and nonfarm economic activities, and labor allocation. This study uses data on labor allocation, daily wage earnings, and food consumption. As each household was comprised of 4.0 persons on average, information on age, education, marital status, rural-urban affiliation, and job status in the sampled seven days were given for all 184,320 members of the sampled 46,080 households. In this study, however, we have only considered the economically employed respondents, who were more than 15 or less than 60 years old. Thus, this study uses the sub-sample consisting of 50,671 individuals, of which 34,152 (67.4%) were from rural areas, 16,519 (32.6%) from urban areas, 6,522 (12.9%) female and 44,149 (87.1%) male.

### 3.2 Models specification and estimation methods

To calculate the economic loss associated with the COVID-19 induced lockdown, we estimate the daily wage earnings (W_i*s*_) of a respondent *i*, working in sector *s* (= farm and nonfarm separately. The wage function is specified as follows:

$$lnW_{is}=\alpha_{1}+\beta_{1s}{\left(Cereal\;price\right)}_{is}+ILC_{is}\phi_{1s}+RLC_{is}\varphi_{1s}+\;\varepsilon_{1s}$$ (1)

where *lnW*_*is*_ is the natural log of daily wage earned by a sampled respondent *i* in sector *s* (= farm *f* or nonfarm *nf*), and *cereal price* is the price of cereals per kg in Bangladesh Taka (BDT/kg). This variable is included in the regression to control for a possible association between cereal price and wage payment of the daily wage workers. The *ILC*_*is*_ is a vector of variables, which includes respondent-level characteristics, such as:

- years of schooling,
- Female dummy (FM) (female = 1, 0 otherwise);
- marital status (married = 1, 0 otherwise);
- age in years;
- a dummy for age group 15–24 years (Age 15–24);
- a dummy for age group 25–34 years (Age 25–34);
- a dummy for age group 35–44 years (Age 35–44);
- a multiplicative dummy consisting of Age 15–24 × FM;
- a multiplicative dummy consisting of Age 25–34 × FM; and
- a multiplicative dummy consisting of Age 35–44 × FM.

The vector of variables *RLC*_*is*_ includes a rural dummy that assumes a value of 1, if a respondent is from a rural area, and 0 otherwise, and 63 dummies for 64 districts of Bangladesh. In addition, in estimating daily wage income, we include inverse Mill’s ratio calculated after estimating Eq (7)- the occupation choice model, to take into account a possible self-selection bias in selecting wage receiving methods of the respondents as will be explained below.

By multiplying the estimated per head average daily wage earnings (in BDT) in the farm sector ${\overset{-}{W}}_{f}$ and that in the nonfarm sector ${\overset{-}{W}}_{nf}$ by the absolute number of daily waged workers in those in Bangladesh, the expected loss of daily wage earnings due to the COVID-19 induced lockdown is calculated as follows:

$$Daily\;income\;loss\;=\;{\bar{W}}_{f}\times N_{f}+{\bar{W}}_{nf}\times N_{nf}.$$ (2)

To estimate the minimum compensation package for the daily wage-based workers to ensure the minimum food security, we specify and estimate a function that explains household level daily food expenditure for the household of daily wage worker *i* (that is, survey respondent *i*) in sector *s*, as follows:

$$lnEDFX_{is}=\alpha_{2}+\gamma_{2s}ln{\bar{DWEH}}_{is}\;+{\left(ILC\right)}_{is}\phi_{2s}+{\left(RLC\right)}_{is}\varphi_{2s}+\;{\left(HLC\right)}_{is}\beta_{2s}+\;\epsilon_{2s},$$ (3)

where *lnEDFX*_*is*_ the natural log of the household level daily total expenditure on all food (in terms of BDT); wage earnings, $ln{\overset{-}{DWEH}}_{is}$ is the natural log of the total daily wage earning of the household of respondent *i* calculated as:

$${\bar{DWEH}}_{is}={\bar{W}}_{is}\times \;\text{No}.\;\text{of}\;\text{earners}\;\text{in}\;\text{the}\;\text{household}\;\text{of}\;\text{respondent}\;i$$ (4)

The definitions of the vectors of variables *ILC*_*is*_ and *RLC*_*is*_ are the same as explained in Eq (1). The vector of variable *HLC*_is_ includes:

- household level income from rent of land and other properties, insurance income, profit and dividend income, lottery and prize money, charity, gift, royalty and assistance both in cash and kind, remittance income, gratuity and retirement benefits, interest received and other income in cash or kind. To make it daily, we divided by 365;
- household level receipt from social safety nets, including any positive income from any social safety net programs by any member of the household. To make it daily, we divided it by 365; and
- land owned (acres).

In estimating Eq (3), we have estimated, firstly without including division dummies; secondly including seven division dummies for eight divisions, and finally including 63 district dummies for 64 districts to capture the regional heterogeneity on the daily household level food expenditure. As both the dependent variable (*lnEDFX*_*is*_), and the estimated household level total daily wage earnings $\left(ln{\overset{-}{DWEH}}_{is}\right)$ are in log form, the coefficient $\hat{\gamma_{2s}}$ in Eq (3) is simply the elasticity. It provides the share of total wage earnings are spent on household level daily food expenditure of a sampled day-labor household in farm and nonfarm sectors. Assuming, zero income of the daily wage-based households in a very strict lockdown situation, the minimum daily support can be calculated as:

$$MS_{s}={\bar{EDFX}}_{is}\times \hat{\gamma_{2s}}$$ (5)

Where ${\overset{-}{EDFX}}_{is}$ is the estimated daily household level total food expenditure in sector *s*.

In reality, however even under a stringent lockdown measure, daily wage earners may desperately search for any miscellaneous work, and thus can earn some positive income (>0). The earnings of a daily wage worker during lockdown time, however, can be lower by a fraction by *θ* than the normal time, where 0 < *θ* < 1. It means, in the lockdown time, the total wage earnings of a daily wage-based household will be $\theta {\overset{-}{DWEH}}_{is}$, which is lower than the normal time household level daily total earnings ${\overset{-}{DWEH}}_{is}$ in Eq (3), where *θ* = 1. Consequently, the logarithm of the household level daily total food expenditure with this lost income ($\theta {\overset{-}{DWEH}}_{is}$) would be smaller than that without income loss by:

$$\begin{array}{l} lnEDFX=f({\bar{DWEH)}}_{is}-lnEDFX=f{\left(\theta \bar{DWEH}\right)}_{is},\\ =\gamma_{2s}ln\left({\bar{DWEH}}_{is}\right)-\gamma_{2s}ln\left(\theta {\bar{DWEH}}_{is}\right)=\gamma_{2s}ln\theta \end{array}$$ (6)

By rearranging Eq (5), we obtain

$$EDFX{\left(\theta \bar{DWEH}\right)}_{is}\;=\;EDFX\;({\left(\bar{DWEH}\right)}_{is}\theta^{\gamma_{2s}}$$ (7)

Where ($\theta {\overset{-}{DWEH})}_{is}$ is the lockdown induced reduced daily total wage earnings of a daily wage-based household, *θ* is the lockdown induced wage earning fraction (0< *θ <* 1), and $\hat{\gamma_{2s}}$ is the estimated coefficient of wage earning in explaining the daily household level total food expenditure *lnEDFX*_*is*_ specified in Eq (3)

A reasonable amount of minimum daily food expenditure support would be the average of the difference between food expenditures with and without income loss due to lockdown measures. Using the estimated coefficient of the daily wage earning $\hat{\gamma_{2s}}$ from Eq (3) and assuming different rates of income share, modifying Eq (5) we recalculate the minimum required daily support to be provided to each household in sector *s* as follows:

$$MS_{s}=1-\theta^{\hat{\gamma 2s}}\times \bar{EDFX{\left(DWEH\right)}_{s}}$$ (8)

where *MS*_*s*_ is the minimum required daily support to a household, $\overset{-}{{EDFX(DWEH)}_{s}}$ is the estimated household level daily food expenditure that is obtained by estimating Eq (3), and *θ* is the estimated coefficient. Eq (8) indicates that the calculated daily minimum support is now contingent upon the fraction of daily wage earning *θ*, the value of $\hat{\gamma_{2s}}$ and the household level daily total food expenditure in the normal time. Under the assumption that a daily wage-based household earns only 10% of their wage income (*θ* = 0.1) in the COVID-19 induced lockdown time compared to the daily wage earning in the normal time, then the household level daily total food expenditure will become fraction 0.1^ ($\hat{\gamma_{2s}}$) of the initial per capita food expenditure, which is the estimated value of the dependent variable of Eq (3). In that case, the minimum support will be 1–0.1^($\hat{\gamma_{2s}}$) *Estimated value of the hosehold level daily food expenditure $(\overset{-}{{EDFX}_{s})}$*.

In suggesting daily minimum support to ensure minimum food security of the daily wage-based households, we assume a range of income loss.

In estimating the wage function (Eq (1)), we have incorporated the inverse Mill’s ratio for controlling the possible self-selection bias due to occupation choice (daily wage-based workers in the farm, or nonfarm sectors. The multinomial logistic regression estimation procedure was employed to characterize the daily wage-based workers in the farm and nonfarm sectors (*i)*, setting the non-daily wage workers as the base (= 0) and assigning a value of 1 for daily wage-based farm workers (N_f_), and a value of 2 for daily wage nonfarm workers (N_nf_) as follows:

$$\begin{array}{l} ln\left(\frac{P\left(daily,\;farm\right)}{P\left(Non\;daily\right)}\right)=\alpha_{30}+HLC_{is}\beta_{3}+\zeta_{3}Homestead_{is}+ILC_{is}\phi_{3}+RLC_{is}\varphi_{3}+\;\varepsilon_{3}\\ ln\left(\frac{P\left(daily,\;nonfarm\right)}{P\left(Non\;daily\right)}\right)=\alpha_{40}+HLC_{is}\beta_{4}+\zeta_{4}Homestead_{is}+ILC_{is}\phi_{4}+RLC_{is}\varphi_{4}+\;\varepsilon_{4} \end{array}$$ (9)

where *HLC*_*is*_, *ILC*_*is*_, and *RLC*_*is*_ are the same as in Eq (2), and *Homestead*_*is*_ is the size of the homestead land owned (acres).

## 4. Results and discussion

Some basic background information of the sampled respondents is presented in Table 2. The sampled respondents are divided into three groups based on the wage payment methods: daily wage workers in farm and nonfarm sectors and other workers. Out of 50,671 sampled respondents, 34,301 (67.7%) were paid other than daily basis mode, 7,552 (14.9%) worked in the farm sector and were paid daily, and 8,818 (17.4%) worked in the nonfarm sector and were paid daily. On average, a sampled respondent was more than 36 years old, with nearly five years of schooling, more than 80% were married, and more than 67% were from rural areas (Table 2). In addition, a sampled household comprised of 4.4 family members, with 1.6 earners (Table 2). Female workers were less likely to be paid on the daily basis. In general, the daily wage workers were mostly engaged in the open field related physical works. In Bangladesh, it is not socially acceptable that females and males perform physical work together in an open field.

**Table 2:** Basic background information of the sampled respondents by their sector of employment.

Payment method	All	Other than daily basis	Daily wage-based workers in		Kruskal-Wallis rank test Chi2 (overall differences)
Sector			Farm	Nonfarm	
		A	b	c	a≠b≠c
No. of respondents	50,671	34,301	7,552	8,818	
Age of the respondents	36.2	35.0^x^	40.1^y^	37.5^z^	1472.7***(0.00)
% Female (FM)	12.9	17.0^x^	4.4^y^	4.2^y^	1586.4***(0.00)
Years of schooling	4.9	5.98^x^	2.2^y^	3.3^z^	5536.7***(0.00)
% Married	81.2	75.9^x^	93.7^y^	90.7^z^	1913.0***(0.00)
% Rural respondents	67.4	63.6^x^	90.1^y^	63.0^x^	2074.3***(0.00)
No. of family members	4.4	4.6^x^	4.1^y^	4.1^y^	839.3***(0.00)
No. of earners	1.61	1.8^x^	1.3^y^	1.3^y^	3597.4***(0.00)
%In age group 15–24 years	17.0	22.1^x^	4.7^y^	7.9^z^	1946.2***(0.00)
%In age group 25–34 years	28.9	28.7^x^	26.1^y^	32.4^z^	81.2***(0.00)
%In age group 35–44 years	26.8	23.8^x^	32.7^y^	33.2^y^	468.8***(0.00)
%From Barishal Division	8.9	9.5^x^	4.6^y^	10.2^z^	202.1***(0.00)
%From Chattogram Division	18.3	19.8^x^	14.8^y^	15.0^y^	177.2***(0.00)
%From Dhaka Division	20.3	22.1^x^	11.9^y^	20.5^z^	403.3***(0.00)
%From Khulna Division	15.2	14.0^x^	19.2^y^	16.6^z^	148.0***(0.00)
%From Mymensingh Division	5.8	5.4^x^	7.5^y^	5.7^x^	50.9***(0.00)
%From Rajshahi Division	12.3	11.5^x^	15.7^y^	12.3^z^	104.1***(0.00)
%From Rangpur Division	12.7	11.0^x^	19.9^y^	12.8^z^	439.2***(0.00)
%From Sylhet Division	6.6	6.7	6.3	6.8	1.63(0.442)

Source: Authors’ based on HIES 2016–17.

*Note*: *** Mean with superscript x is statistically significantly different from mean with y or z in the same line at the 1% level of alpha error probability, based on multiple Mann-Whitney tests accounting for family-wise error; *P*-values in parentheses.

A closer scrutiny of Table 2 reveals that, on average, the daily wage-based workers in the farm sector are statistically significantly older (40.1 years) with significantly fewer years of schooling (2.2 years) and was more likely to be from rural areas compared to others. In addition, the number of family members and earners were lower for households of daily wage workers in both farm and nonfarm sectors compared to the other group. Thus, daily wage-based workers in general, are relatively more resource poor compared to other workers.

More than one-fifth of the sampled respondents were from Dhaka Division, followed by Chattogram (18.3%) and Khulna Divisions (15.2%) (Table 2). Dhaka and Chattogram are the largest garment industry clusters in Bangladesh [58], where more than 4.4 million workers are employed, the majority of which are female [47]. Thus, as there are more nonfarm work opportunities in Dhaka and Chattogram than other Divisions of Bangladesh, the largest share of sampled respondents was drawn from these two Divisions. Nearly 20% of the daily wage-based farmworkers were from Rangpur Division (Table 2). Of the total population of Bangladesh, 23.4% live below the national poverty line [57]. Rangpur Division has the highest incidence of income poverty (30.5%) [57], which is reflected in the concentration of daily waged-based farmworkers.

As per Table 3, more than 13% of respondents did not have their own homestead land, and more than 11% were landless. On average, the daily wage earning in the farm sector was BDT 314, and BDT 421 in the nonfarm sector (Table 3). The yearly average income from land rent, rent of other properties, insurance, profits and dividends, lottery and prize money, charity, gifts, royalties and other assistance both in cash and kind, remittances, gratuities and retirement benefits, interest received and other income in cash or kind, was BDT 11,427. However, this income was significantly lower for daily wage workers in the farm sector (BDT 4,661.6) and the nonfarm sector (BDT 5,343.5). More than 5% of respondents reported inclusion in the social safety net programs, with the yearly average receipt from them being nearly BDT 10,414. It shows that daily wage workers were more likely to be included in a social safety net program than others (Table 3).

**Table 3:** Income, characteristics and food expenditure of the sampled respondents by their sector of employment.

			Daily wage-based labor in		
Sector	All	Salaried worker	Farm	Nonfarm	Kruskal-Wallis rank test Chi2 (overall differences)
Variables		a	b	c	a≠b≠c
%With no homestead land	13.2	12.3^x^	11.5^y^	18.1^z^	226.3***(0.00)
Size of the homestead land/dwelling area (acres)	0.12	0.13^x^	0.09^y^	0.08^z^	1120.4***(0.00)
%Landless	11.1	10.2^x^	9.6^x^	15.9^y^	249.4***(0.00)
Total land owned (acres)	0.74	0.89^x^	0.62^y^	0.25^z^	2116.2***(0.00)
Daily wage earning (BDT)^d^			313.8	420.6	-106.9*** (-9.44)
Yearly total income from remittances, property income, pension, gratuity and gifts (BDT)	11,427.0	14,480.5^x^	4,661.6^y^	5,343.5^z^	489.3***(0.00)
%Included in any social safety net program	5.3	3.4^x^	9.4^y^	8.9^y^	717.9***(0.00)
Yearly total received from social safety net programs (BDT)	10,413.9	6,698.4^x^	19,007.3^y^	17,507.0^y^	719.8***(0.00)
Daily household level total expenditure on all food (BDT)	213.5	231.2^x^	165.2^y^	186.4^z^	2945.2***(0.00)
Daily household level total consumption of cereals (kg)	1.80	1.83	1.81	1.6^1^	459.3***(0.00)
Daily household level total expenditure on cereals only (BDT)	64.7	67.1^x^	61.3^y^	58.0z	587.7***(0.00)
Price of cereals (BDT/kg)	37.4	37.7^x^	34.3^y^	36.8^z^	1225.8***(0.00)

Source: HIES 2016–17.

*Note*:

*** Mean with superscript x is statistically significantly different from mean with y or z in the same line at the 1% level of alpha error probability, based on multiple Mann-Whitney tests accounting for family-wise error; *P*-values in parentheses.

^d^For daily wage earnings, the t-test is applied to examine the mean differences of the wage earning between farm and nonfarm sectors. Differences = Mean (b)–Mean (c). H0: Diff = 0, Ha: Diff < 0 (one-sided t-test).

The average daily household level total food expenditure was BDT 213.5, of which the expenditure only on cereals accounted for 30% (BDT 64.7). The daily per household level consumption of cereals was 1.8kg (Table 3). This indicates the importance of cereals as a cheap source of dietary energy for the poor in developing countries. Importantly, as expected, the daily household level total food expenditure was significantly lower for daily wage farmworkers, which confirms that the daily wage-based workers are more distressed than others.

Tables 4–5 present estimated functions specified in Eqs (1) and (3). Table 4 presents the estimated functions explaining the (ln) daily earning per earner for farm and nonfarm workers. The estimates were obtained by means of the Generalized Linear Model (GLM) estimator. There is a positive relationship between years of schooling and the daily wage earnings in the nonfarm sector, however, such relation is insignificant in the case of the farm sector (Table 4). On average, the female daily wage workers were paid less than their male counterparts in both farm and nonfarm sectors (Table 4). The coefficients on the district dummies can be seen in S1 Table. The estimated daily wage earnings for farm workers is BDT 272.2, and BDT 361.5 for nonfarm workers (Table 4).

**Table 4:** Estimated functions explaining the (ln) daily wage earnings of the farm and nonfarm workers: Generalized Linear Model (GLM) estimates.

Sector	Farm	Nonfarm
Dependent variable	Ln (daily wage earning (cash and the monetary value of the in kind receipt) received on daily basis)	
Independent variables		
Price of cereals (BDT/kg)	0.00001 (0.00)	0.001 (0.00)
Years of schooling	0.001 (0.00)	0.008*** (0.00)
Married dummy (yes = 1)	0.04 (0.02)	0.18*** (0.03)
Female dummy (yes = 1)	-0.56*** (0.05)	-0.58*** (0.07)
Age	-0.001 (0.00)	0.0002 (0.00)
Age group 15–24 dummy (yes = 1) (Age 15–24)	-0.03 (0.05)	-0.021 (0.05)
Age group 25–34 dummy (yes = 1) (Age 25–34)	0.01 (0.03)	0.029 (0.04)
Age group 35–44 dummy (yes = 1) (Age 35–44)	0.02 (0.02)	0.07*** (0.03)
FM × Age 15–24	0.06 (0.14)	-0.29 (0.20)
FM × Age 25–34	0.23** (0.10)	-0.15 (0.12)
FM × Age 35–44	0.15** (0.07)	0.08 (0.12)
Rural dummy (yes = 1)	-0.04 (0.02)	-0.09*** (0.01)
Districts effects are controlled by including 63 district dummies for 64 districts (S1 Table)		
Inverse Mills ratio	-0.011 (0.01)	-0.052*** (0.01)
Constant	5.74*** (0.11)	5.52*** (0.11)
No. of observations	7552	8818
AIC	0.82	1.26
BIC	-65774.59	-77611.62
Log likelihood	-3016.2	-5497.51
Estimated daily wage earnings per earner (BDT) (w)	272.2	361.5
No. of earners (e)	1.30	1.31
Daily total wage earned (BDT) {w × e}	353.9	473.6

Notes: Values in parentheses are robust standard errors calculated applying bootstrap method replicating estimation 1000 times.

***, ** and * indicate the 1% level, 5% level and 10% level of significance, respectively.

**Table 5:** Estimated functions explaining the (ln) household level daily total food expenditure (BDT) of the daily wage workers in farm and nonfarm sectors: Ordinary Least Square (OLS) estimates.

Dependent variable		Ln (household level daily total expenditure on all food (BDT)				
Sector	Farm			Nonfarm		
Independent variables	Model (1)	Model (2)	Model (3)	Model (4)	Model (5)	Model (6)
Estimated ln (daily total wage earnings (BDT))	0.34*** (0.01)	0.20*** (0.01)	0.23*** (0.01)	0.41*** (0.01)	0.30*** (0.01)	0.29*** (0.01)
Daily income from pension, gratuity, grants, remittance (BDT)	0.0003** (0.00)	0.0003** (0.00)	0.0003** (0.00)	0.00053*** (0.00)	0.00045*** (0.00)	0.00046*** (0.00)
Daily received from social safety net programs (BDT)	-0.00002* (0.00)	-0.00002 (0.00)	-0.00001 (0.00)	-0.000023** (0.00)	-0.00003*** (0.00)	-0.000014 (0.00)
Years of schooling	0.006*** (0.00)	0.005*** (0.00)	0.005*** (0.00)	0.0057*** (0.00)	0.0073*** (0.00)	0.0085*** (0.00)
Total operating land (acres)	0.002* (0.00)	0.001 (0.00)	0.002 (0.00)	-0.0017 (0.01)	-0.0020 (0.01)	-0.0020 (0.01)
Dummy for the rural household (yes = 1)	-0.04** (0.02)	-0.04** (0.02)	-0.03* (0.02)	-0.0081 (0.01)	-0.026** (0.01)	-0.020* (0.01)
Female dummy (yes = 1)	-0.43*** (0.03)	-0.49*** (0.03)	-0.46*** (0.03)	-0.37*** (0.03)	-0.42*** (0.03)	-0.42*** (0.03)
Age	0.003*** (0.00)	0.004*** (0.00)	0.004*** (0.00)	0.0031*** (0.00)	0.0047*** (0.00)	0.0054*** (0.00)
Division dummy (Barishal = 0)			Districts effects are controlled by including 63 district dummies for 64 districts (S2 Table)			Districts effects are controlled by including 63 district dummies for 64 districts (S2 Table)
Chattogram division (yes = 1)		0.10*** (0.03)			0.21*** (0.02)	
Dhaka division (yes = 1)		0.099*** (0.03)			0.096*** (0.02)	
Khulna division (yes = 1)		-0.11*** (0.02)			-0.079*** (0.02)	
Mymensingh division (yes = 1)		-0.091*** (0.03)			-0.095*** (0.02)	
Rajshahi division (yes = 1)		-0.21*** (0.02)			-0.16*** (0.02)	
Rangpur division (yes = 1)		-0.21*** (0.02)			-0.19*** (0.02)	
Sylhet division (yes = 1)		0.13*** (0.03)			0.27*** (0.03)	
Constant	2.96*** (0.08)	3.77*** (0.09)	3.43*** (0.09)	2.46*** (0.08)	3.09*** (0.08)	3.08*** (0.09)
No. of observations	7552	7552	7552	8818	8818	8818
R-squared	0.16	0.22	0.33	0.19	0.27	0.35
Wald chi2(8,15)	1319.36	2005.10	3756.29	1884.35	2835.95	5337.26
Prob > chi2	0.00	0.00	0.00	0.00	0.00	0.00
Expected daily household level expenditure on food (BDT)	149.5	150.6	152.5	167.2	168.84	170.8

Notes: Values in parentheses are robust standard errors calculated applying bootstrap method replicating estimation 1000 times.

***, ** and * indicate the 1% level, 5% level and 10% level of significance, respectively.

In 2016–17, there were 24.7 million workers in the farm sector and 36.1 million in the nonfarm sector (Table 1). There is no straightforward information on the share of workers who were paid on daily basis. In 2016–17, however, 44.4% workers in the rural area, and 18.5% workers in the urban area were paid on daily basis [53]. As most of the workers in the rural area of Bangladesh are mainly engaged in farming, we assume that out of 24.7 million workers in the farm sector, 44.7% of them, or 10.9 million were paid on daily basis. Similarly, as in the urban area, most of the workers are mostly engaged in the nonfarm sector, we assume that out of 36.1 million workers in the nonfarm sector, 18.5% of them, or 6.7 million, were paid on daily basis. Following Eq (2), multiplying the estimated daily wage earnings for farmworkers as BDT 272.2/day and for the nonfarm worker as BDT 361.5/day, under the assumption of a complete lockdown with no-one allowed to work, the economic loss in one day is estimated at BDT 5389.03 million or approximately US$ 64.2 million. Assuming 50% of the daily wage workers are not allowed to work and the rest are, the economic loss/day will be BDT 2694.5 million or US$ 32.1 million.

Table 5 presents the estimated functions applying the Ordinary Least Square (OLS) estimation approach, explaining the (ln) daily household level total food expenditure of the sampled farm and nonfarm daily wage-based households. We have presented the results of three different models. In Model (1), we did not include the Division or district dummies, whereas in Model (2) we included seven Division dummies for eight divisions; and in Model (3) we included 63 district dummies for 64 districts.

In the estimated functions, we have included the natural log (ln) of the estimated daily total wage earnings of a sampled household in explaining the daily household level total food expenditure of the sampled daily wage-based farm and nonfarm households (Table 5). The estimated (ln) daily household level total wage earnings are highly statistically significant and positive in explaining the (ln) daily household level total expenditure on food (Table 5). It shows that a 1% increase in total household level daily wage earnings leads to an increase in the daily household level food expenditure by 0.2% (Model 2) at the minimum to 0.34% at the maximum for the daily wage-based farm households in the farm sector, and a 1% increase in wage earnings leads to an increase in the daily per capita food expenditure by 0.29% (Model 6) to 0.41% (Model 4) at the maximum for the daily wage-based nonfarm households (Table 5). The income from other sources, years of schooling and age also positively and significantly affect the daily per capita food expenditure of the sampled farm and nonfarm workers. This shows that, on average, both farm and nonfarm daily wage-based households in Khulna, Mymensingh, Rangpur and Rajshahi Divisions spend statistically significantly less on food per capita compared to the households in Barishal Division, which is the base Division. The coefficients on the district dummies can be seen in S2 Table.

In Table 5, in the case of the sampled daily wage-based household in the farm sector, the estimated coefficient of the daily total wage earnings in the household level daily total food expenditure $(\hat{\gamma_{2f})}$ are 0.34, 0.20 and 0.23, respectively (Models 1–3). The estimated daily household level food expenditures $(\overset{-}{{EDFX}_{f})}$ are BDT149.5, BDT150.6 and BDT152.5, respectively (Models 1–3). Similarly, the estimated share of the daily total wage earnings in the household level daily total food expenditure $(\hat{\gamma_{2f})}$ are 0.41, 0.30 and 0.29, respectively (Models 4–6), and the estimated daily household level food expenditures $(\overset{-}{{EDFX}_{nf})}$ are BDT167.2, BDT168.8, and BDT170.8 respectively (Models 4–6). Assuming 10%, 20% and 30% of the daily earnings of the daily wage-based households in the farm and nonfarm sectors (*θ* = 0.1, 0.2 *and* 0.3) compared to the normal time daily wage earnings, the estimated daily minimum supports are presented in Table 6. In estimating the daily minimum support, we have set γ_2*f*_ = 0.34 and $\overset{-}{{EDFX}_{f}}=$ BDT 152.5 for the daily wage-based farm household, and *γ*_2*nf*_ = 0.41 and $\overset{-}{{EDFX}_{nf}}=$ BDT 178.5, for the daily wage-based nonfarm households. Our estimation shows that the estimated daily minimum support is ranged from BDT 51.2–83 depending on the share of the daily wage earnings (*θ*) for the daily wage-based farm households, and it ranged between BDT63-104 in the case of the daily wage-based household in the nonfarm sector under the assumptions of 70%, 80% and 90% income loss (*θ* = 0.1, 0.2 *and* 0.3) due to COVID-19 induced lockdown time (Table 6). The estimation suggests a common minimum support at US $ 1 per daily wage-based household in Bangladesh to ensure minimum food security during COVID-19 induced lockdown time. However, our estimation process is flexible which contingent upon the share of income loss due to the severity of the lockdown. This flexibility in estimating the daily minimum support will allow policymakers to set the daily minimum support based on the severity of a lockdown situation in a specific region.

**Table 6:** Calculation of minimum daily support using the estimated daily household level food expenditure and the estimated coefficient $(\hat{\gamma_{2})}$ reported in Table 5.

	Farm sector			Nonfarm sector		
Assumed income share during COVID-19 induced lockdown (*θ*)	Estimated coefficient of $(\hat{\gamma_{2})}$	Estimated daily household level food expenditure	Suggested daily minimum support (BDT)	Estimated coefficient of $(\hat{\gamma_{2})}$	Estimated daily household level food expenditure	Suggested daily minimum support (BDT)
0.1	0.34	152.5	82.8	0.41	170.8	104.
0.2	0.34	152.5	64.3	0.41	170.8	82.5
0.3	0.34	152.5	51.2	0.41	170.8	66.5

Source: Authors’ based on Table 5.

Table 7 presents the estimated functions that characterize the sampled daily wage workers (Eq 9), applying the multinomial logit estimation procedure setting the workers who receive their remuneration, not on a daily basis as the base (= 0). Compared to the base workers, respondents with higher income, with more family members and more years of school and female respondents were less likely to be daily wage-based workers (Table 7). In contrast, married and rural respondents; and relatively older respondents, were more likely to be daily wage-based workers both in farm and nonfarm sectors. Female respondents were in general less likely to be daily wage-based workers, and specifically, female respondents in age groups 15–24, 25–34, and 35–44 years old were less likely to work as daily wage-based workers in both farm and nonfarm sectors. In characterizing the daily wage-based workers, the district level effects are also included by including 63 dummies for 64 districts setting Bagerhat district as the base (= 0). The coefficients of the district dummies can be seen in S3 Table. After estimating the functions that characterize the sampled daily wage-based workers reported in Table 7, the generalized inverse Mill’s ratios are calculated separately for daily wage-based workers in farm and nonfarm sectors following Vella’s [59] procedure, and plugged in into Eq (1) in estimating daily wage earnings (Table 4).

**Table 7:** Estimated function explaining the occupation choice of the sampled respondents: Multinomial logit estimates with base category being salaried workers (= 0).

Dependent variable: Daily basis worker	Farm sector (yes = 1)		Nonfarm sector (yes = 2)	
Estimation procedure	Multinomial logit	Marginal effects (dy/dx delta method)	Multinomial logit	Marginal effects (dy/dx delta method)
Independent variables				
Size of the homestead land owned (acres)	-0.14 (0.10)	-0.01 (0.01)	-0.083 (0.10)	-0.01 (0.01)
Daily income from pension, gratuity, grants, remittance (BDT)	-0.0020*** (0.00)	-0.0001** (0.00)	-0.0025*** (0.00)	-0.0003*** (0.00)
Daily received from social safety nets program (BDT)	0.00024 (0.00)	0.00002 (0.00)	0.00023 (0.00)	0.00002 (0.00)
No. of family members	-0.28*** (0.01)	-0.02*** (0.00)	-0.24*** (0.02)	-0.02*** (0.00)
Years of schooling	-0.24*** (0.01)	-0.02*** (0.00)	-0.18*** (0.01)	-0.02 (0.00)
Married dummy (yes = 1)	0.65*** (0.08)	0.04*** (0.007)	0.58*** (0.08)	0.05 (0.01)
Female dummy (yes = 1) (FM)	-1.52*** (0.11)	-0.11*** (0.01)	-1.00*** (0.19)	-0.08*** (0.02)
Age	0.0070 (0.01)	0.0003 (0.00)	0.011* (0.01)	0.001 (0.00)
Age group 15–24 dummy (yes = 1) (Age15-24)	-1.04*** (0.19)	-0.09*** (0.02)	-0.12 (0.21)	0.02 (0.03)
Age group 25–34 dummy (yes = 1) (Age25-34)	0.12 (0.13)	-0.01 (0.01)	0.64*** (0.15)	0.08*** (0.02)
Age group 35–44 dummy (yes = 1) (Age35-44)	0.30*** (0.08)	0.01 (0.01)	0.62*** (0.10)	0.07 (0.01)
FM × Age15-24	-0.90* (0.47)	-0.005 (0.04)	-2.47*** (0.46)	-0.29*** (0.06)
FM × Age25-34	-0.74*** (0.23)	-0.01 (0.02)	-1.84*** (0.24)	-0.21 (0.03)
FM × Age35-44	-0.63*** (0.17)	-0.02 (0.02)	-1.23*** (0.26)	-0.14 (0.03)
Total operating land (acres)	-0.025 (0.02)	0.001 (0.01)	-0.11 (0.22)	-0.01 (0.03)
Rural dummy (yes = 1)	1.18*** (0.06)	0.11*** (0.00)	-0.18*** (0.06)	-0.06*** (0.01)
Districts effects are controlled by including 63 district dummies for 64 districts (S1 Table)				
Constant	-0.54* (0.32)		0.46 (0.36)	
Calculated inverse Mill’s ratio (mean)	-3.732451		-3.93767	
No. of observations	50671			
Wald chi2(158)	177460.12			
Prob > chi2	0.00			
Pseudo R2	0.22			
Log pseudolikelihood	-28518766			

Notes: Values in parentheses are robust standard errors.

***, ** and * indicate the 1% level, 5% level and 10% level of significance, respectively.

It is noted that the methodology of this study is simple and relatively easily replicable for other countries. For example, in India, 34.5% of employed workers are casual labor, of which 21% are engaged in agriculture [60]. The daily average wage earnings per day by a casual labor ranged between India Rupee (Rs.) 253–282 [60]. A recent report stressed that in India, in April 2020, 12 million people became jobless, and the unemployment rate had increased from 8.8% in March 2020 to 23.5% in April 2020, due to COVID-19 induced turmoil [61]. By examining the share of expenditure of the daily income on food, it is possible to suggest a minimum support package to ensure the food security of casual labor-based households in India during the COVID-19 induced lockdown period.

## 5. Conclusion and policy implications

Using information of more than 50,000 respondents from the HIES 2016–17 dataset, this study, firstly quantified the economic loss due to the COVID-19 induced lockdown and suggested the minimum support package to ensure food security of the daily wage-based workers in Bangladesh. Nearly half of the world’s population is now under some form of restrictions imposed by national governments to curb the spread of COVID-19. While this lockdown may restrict the spread of COVID-19, in the absence of effective support, it may also generate severe food and nutrition insecurity for daily wage-based workers, particularly in the developing countries [10, 27]. The ILO warned that due to COVID-19 induced movement restrictions and lockdown, the participation of the global working force in the first quarter of 2020 has declined by 4.5% which is equivalent to 130 million full-time jobs [8].

In Bangladesh, out of a 60.8 million employed labor force, 24.7 million (40.6%) are directly engaged in the farm sector, of which 44.4% are paid on daily basis, and 36.1 million are engaged in the nonfarm sector, of which 18.4% are paid on daily basis. This study stressed that, compared to others, the daily wage-based workers in both farm and nonfarm sectors are comparatively more resource poor in terms of land ownership and education. Based on the estimated daily wage earnings, this study found that under the assumption of complete lockdown, the daily economic loss due to prohibiting daily wage-based workers to work would be US$ 64.2 million.

This study also demonstrated that the average wage elasticity in explaining the daily food expenditure at the household level ranged from 0.20 to 0.34, and the daily household food expenditure ranged from BDT 149.5–152.5 for the daily wage-based farm households. For the daily wage-based nonfarm households, the average wage elasticity in explaining the daily food expenditure at the household level ranged from 0.29 to 0.41, and the daily household food expenditure ranged from BDT 167–171. Assuming 10%, 20% and 30% of the daily earnings of the daily wage-based households in the farm and nonfarm sectors (*θ* = 0.1, 0.2 *and* 0.3) compared to the normal time income, we have estimated that on average it is necessary to provide daily BDT 51–104 or around US $ 1 per daily wage-based households during the COVID-19 induced lockdown time. It is important to mention here is that, the suggested minimum support US$ 1/day/household is calculated based on considering only food expenditure. The suggested minimum support package is thus only suitable for the short-term. In the case of a long-term lockdown situation, it is necessary to include the costs of health, education, clothing and housing.

Recently, the government of Bangladesh announced the provision of approximately US$ 24/month to two million to households [62]. In addition, 20kg cereals and food will be provided to 1 million households in Bangladesh [63]. While the amount of support is in line with our findings, in the case of a lengthy lockdown period, it is necessary to consider other household costs, such as clothing, medicine and education in the support package. Moreover, as the labor market of Bangladesh is comprised of 60.8 million active workers, the coverage of the safety net should be expanded in the case of a lengthier lockdown. As food and nutrition insecurity can have long-run impacts on human capital formation, the government must expand the emergency safety network program to include almost all marginal households or consider loosening restrictions in the agriculture sector. Without effective support programs, an implementation of a strict lockdown for a long time may be very difficult, if the poor households are forced to come out to search for works, money and food. In such case, to support smallholder agriculture, wage workers and agricultural value chains, the government should consider issuing movement passes to persons and carriers of agricultural input and output in the case of a very strict lockdown scenario. Finally, it is also imperative to take necessary steps against government failure in the form of leakage in distributing compensation packages. Otherwise, the benefits of government effort may not reach to the vulnerable groups.

## Supporting information

S1 Table District dummies included in explaining daily wage earnings (reported in Table 4) of the daily wage workers in the farm and nonfarm sectors (Bagerhat district is the base = 0). (DOCX)

S2 Table District dummies included in explaining the daily household level total food expenditure (reported in Table 5). Base district: Bagerhat = 0.
(DOCX)

S3 Table District dummies included in explaining the occupation choice (reported in Table 7). Base district: Bagerhat.
(DOCX)

S1 File (DO)

S2 File (DTA)

## References

[1] WHO. Coronavirus disease (COVID- 19) Pandemic: Public advice. In: World Health Organization (WHO) [Internet]. 2020 [cited 19 Aug 2020]. Available: https://www.who.int/emergencies/diseases/novel-coronavirus-2019

[2] Schumaker E. Timeline: How Coronavirus Got Started: The outbreak spanning the globe began in December, in Wuhan, China. ABC News. 2020: 1–9. Available: https://abcnews.go.com/Health/timeline-coronavirus-started/story?id=69435165

[3] WHO. Coronavirus (COVID-19) events as they happen. In: World Health Organization (WHO): Updated 9 May, 2020. 2020 pp. 1–156.

[4] Li Q, Guan X, Wu P, Wang X, Zhou L, Tong Y, et al Early Transmission Dynamics in Wuhan, China, of Novel Coronavirus–Infected Pneumonia. N Engl J Med. 2020;382: 1199–1207. 10.1056/NEJMoa2001316 31995857 PMC7121484

[5] Wu JT, Leung K, Leung GM. Nowcasting and forecasting the potential domestic and international spread of the 2019-nCoV outbreak originating in Wuhan, China: a modelling study. Lancet. 2020;395: 689–697. 10.1016/S0140-6736(20)30260-9 32014114 PMC7159271

[6] He X, Lau EHY, Wu P, Deng X, Wang J, Hao X, et al Temporal dynamics in viral shedding and transmissibility of COVID-19. Nat Med. 2020;26 10.1038/s41591-019-0732-8 32296168

[7] Dunford D, Dale B, Stylianou N, Lowther E, Ahmed M, delaTorre Arena I. Coronavirus: The world in lockdown in maps and charts How the world shut down. In: BBC News [Internet]. 2020 Available: https://www.bbc.com/news/world-52103747

[8] ILO. ILO Monitor: COVID-19 and the world of work. Third edition Updated estimates and analysis. Geneva; 2020. Available: https://www.ilo.org/wcmsp5/groups/public/@dgreports/@dcomm/documents/briefingnote/wcms_743146.pdf

[9] IMF. World Economic Outlook, Chapter 1: The Great Lockdown. Int Monet Fund. Washington D.C.; 2020. Available: https://www.imf.org/en/Publications/WEO/Issues/2020/04/14/weo-april-2020

[10] FSIN. 2020 Global Report on Food Crises: Joint Analysis for Better Decisions. Rome; 2020. Available: https://docs.wfp.org/api/documents/WFP-0000114546/download/

[11] Harvey F. Coronavirus measures could cause global food shortage, UN warns. The Guardian. 26 3 2020: 1–6. Available: https://www.theguardian.com/global-development/2020/mar/26/coronavirus-measures-could-cause-global-food-shortage-un-warns

[12] Hamilton PC, Nkurunziza J. COVID-19 and food security in vulnerable countries. In: United Nations Conference on Trade and Development [Internet]. 2020 [cited 11 May 2020] pp. 1–5. Available: https://unctad.org/en/pages/newsdetails.aspx?OriginalVersionID=2331

[13] World Bank. Food Security and COVID-19. In: World Bank Policy Brief [Internet]. 2020 [cited 12 May 2020] pp. 1–7. Available: https://www.worldbank.org/en/topic/agriculture/brief/food-security-and-covid-19

[14] Mengoup FE. Ensuring food security during the Covid-19 pandemic. Rabat (Morocco); 2020. Report No.: PB 20–26. Available: https://www.policycenter.ma/sites/default/files/PB-20–26(FMEngoub).pdf

[15] Schmidhuber J, Pound J, Qiao B. COVID-19: Channels of transmission to food and agriculture. Rome; 2020. Available: http://www.fao.org/3/ca8430en/CA8430EN.pdf

[16] Readon T, Bellemare MF, Ziliberman D. How COVID-19 may disrupt food supply chains in developing countries. International Food Policy Research Institute. Washington D.C.: International Food Policy Research Institute (IFPRI); 2020. pp. 1–6. Available: https://www.ifpri.org/blog/how-covid-19-may-disrupt-food-supply-chains-developing-countries

[17] Dev BYSM. Addressing COVID-19 impacts on agriculture, food security, and livelihoods in India. Washington D.C.: International Food Policy Research Institute (IFPRI); 2020. pp. 1–5. Available: https://www.ifpri.org/blog/addressing-covid-19-impacts-agriculture-food-security-and-livelihoods-india

[18] Hossain ST. Impacts of COVID-19 on the Agri-food Sector: Food Security Policies of Asian Productivity Organization Members. J Agric Sci—Sri Lanka. 2020;15: 116–132.

[19] Roser M. The short history of global living conditions and why it matters that we know it. In: Our World In Data [Internet]. 2019 [cited 7 Nov 2019] pp. 1–6. Available: https://ourworldindata.org/a-history-of-global-living-conditions-in-5-charts?linkId=62571595

[20] WHO. Global hunger continues to rise, UN report says. In: World Health Organization (WHO) [Internet]. Rome; 2018 [cited 21 Oct 2019] pp. 1–9. Available: https://www.who.int/news-room/detail/11-09-2018-global-hunger-continues-to-rise—new-un-report-says

[21] FAO, IFAD, UNICEF, WFP, WHO. The State of Food Security and Nutrition in the World 2017: Building Resilience for Peace and Food Security. Rome: Food and Agriculture Organization of the United Nations; 2017. Available: http://www.fao.org/3/a-i7695e.pdf

[22] World Bank. Poverty and Shared Prosperity 2018: Piecing Together the Poverty and Puzzle. Washington D.C.: World Bank; 2018 Available: https://openknowledge.worldbank.org/bitstream/handle/10986/30418/9781464813306.pdf

[23] United Nations. Sustainable Development Goals 2: Zero Hunger. In: Sustainable Development Goals [Internet]. 2020 [cited 12 May 2020] pp. 1–7. Available: https://www.un.org/sustainabledevelopment/hunger/

[24] Abiad A, Arao M, Lavina E, Platitas R, Pagaduan J, Jabaga C. The impact of COVID-19 on developing Asian economies: The role of outbreak severity, containment stringency, and mobility declines In: Djankov S, Panizza U, editors. COVID-19 in Developing Economies. London: CEPR Press, Centre for Economic Policy Research, 33 Great Sutton Street London, EC1V 0DX, UK; 2020 pp. 86–99. Available: https://voxeu.org/content/covid-19-developing-economies

[25] IMF. World Economic Outlook Update June 2020: A crisis like no other, an uncertain recovery. Int Monet Fund. Washington D.C.; 2020. Available: https://www.imf.org/en/Publications/WEO/Issues/2020/06/24/WEOUpdateJune2020

[26] ECLAC and ILO. Work in times of pandemic: the challenges of the coronavirus disease (COVID-19): Employment Situation in Latin America and the Caribbean. Santiago (Chile) and Geneva; 2020. Report No.: No. 22 (LC/TS.2020/46). Available: https://www.cepal.org/en/publications/45582-employment-situation-latin-america-and-caribbean-work-times-pandemic-challenges

[27] WFP. COVID-19 will double number of people facing food crises unless swift action is taken. In: World Food Programme [Internet]. 2020 [cited 11 May 2020] pp. 3–5. Available: https://www.wfp.org/news/covid-19-will-double-number-people-facing-food-crises-unless-swift-action-taken

[28] Fan S. Preventing global food security crisis under COVID-19 emergency. Washington D.C.: International Food Policy Research Institute (IFPRI); 2020 pp. 1–5. Available: https://www.ifpri.org/blog/preventing-global-food-security-crisis-under-covid-19-emergency

[29] FAOSTAT. Producer prices- Annual. Faostat. Rome: Food and Agriculture Organization of the United Nations (FAO); 2020. pp. 1–5. Available: http://www.fao.org/faostat/en/#data/PP

[30] Mottaleb KA, Rahut DB, Erenstein O. Do market shocks generate gender-differentiated impacts? Policy implications from a quasi-natural experiment in Bangladesh. Womens Stud Int Forum. 2019;76: 102272 10.1016/j.wsif.2019.102272 31853162 PMC6894339

[31] Mallick D. Are Female-Headed Households More Food Insecure? Evidence from Bangladesh. World Dev. 2010;38: 593–605. 10.1016/j.worlddev.2009.11.004

[32] Balagtas J V. Did the commodity price spike increase rural poverty? Evidence from a long-run panel in long-run panel in Bangladesh. Agric Econ. 2014;45: 303–312. 10.1111/agec.12066

[33] Laborde D, Abdullah M, Parent M. Tracking Government Responses Affecting Global Food Markets during the COVID-19 Crisis. Washington D.C.; 2020. Report No.: 1. Available: http://ebrary.ifpri.org/utils/getfile/collection/p15738coll2/id/133711/filename/133921.pdf

[34] United Nations. World Economic Situation And Prospects. COVID-19 Disrupting lives, Econ Soc. New York; 2020. Report No.: April 2020 Briefing No. 136. Available: https://www.un.org/development/desa/dpad/publication/world-economic-situation-and-prospects-april-2020-briefing-no-136/

[35] Sumner A, Hoy C, Ortiz-juarez E. Estimates of the impact of COVID-19 on global poverty. Helsinky; 2020. Report No.: WIDER Working Paper 2020 / 43. Available: https://www.wider.unu.edu/sites/default/files/Publications/Working-paper/PDF/wp2020-43.pdf

[36] FAO, IFAD, UNICEF, WFP, WHO. The State of Food Security and Nutrition in the World 2019. Safeguarding against economic slowdowns and downturns. Rome: Food and Agriculture Organization of the United Nations (FAO); 2019. 10.1109/JSTARS.2014.2300145

[37] Worldmeter. Coronavirus Cases: Bangladesh. In: COVID-19 for Coronavirus Pendamic [Internet]. 2020 [cited 13 May 2020] pp. 1–8. Available: https://www.worldometers.info/coronavirus/country/bangladesh/

[38] Government of Bangladesh. COVID-19: Bangladesh Multi-Sectoral Anticipatory Impact and Needs Analysis. Dhaka; 2020. Available: https://reliefweb.int/sites/reliefweb.int/files/resources/COVID_NAWG Anticipatory Impacts and Needs Analysis.pdf

[39] The Daily Prothom Alo. The Holiday has Increased to May 30, 2020, Strict Measures in Eid. 2020: 1–2. Available: https://www.prothomalo.com/bangladesh/article/1656388/ছুটি-বাড়ছে-৩০-মে-পর্যন্ত-ঈদে-কড়াকড়ি

[40] The Business Standard. COVID-19 in Bangladesh: 55 districts under partial or complete lockdown. COVID-19 in Bangladesh. 17 Apr 2020: 1–5. Available: https://tbsnews.net/coronavirus-chronicle/covid-19-bangladesh/48-districts-under-partial-or-complete-lockdown-70663

[41] World Bank. World Development Indicators. In: Data Bank, World Development Indicators [Internet]. 2020 [cited 7 Feb 2019] p. 64168445. Available: https://databank.worldbank.org/data/reports.aspx?source=world-development-indicators#

[42] Quasem MA. Conversion of Agricultural Land to Non-agricultural Uses in Bangladesh: Extent and Determinants. Bangladesh Dev Stud. 2011;34: 59–85.

[43] FAO. Data: Production. In: Online database on crop production,yield and harvested area [Internet]. Rome; 2020 [cited 26 Apr 2020] pp. 1–5. Available: http://www.fao.org/faostat/en/#data/QC

[44] FAO. Food Balance Sheets: Report. In: Online database on food balance sheets [Internet]. 2019 [cited 19 Aug 2019] pp. 1–6. Available: http://www.fao.org/faostat/en/#data/FBS

[45] The Economist. Global Food Security Index 2019. In: Global Food Security Index 2019: Strengthening food systems and the environment through innovation and investment, The Economist and Intelligence Unit [Internet]. 2019 [cited 13 May 2020]. Available: https://foodsecurityindex.eiu.com/Index/Overview

[46] Wiesmann DD, Weingärtner DL, Schöninger DI. The Challenge of Hunger Global Hunger Index: Facts, determinants, and trends. Food Policy. 2006.

[47] Fathi N. Safety First: Bangladesh Garment Industry Rebounds. In: International Finance Corporation Insights [Internet]. 2020 [cited 19 May 2020] pp. 1–5. Available: https://www.ifc.org/wps/wcm/connect/news_ext_content/ifc_external_corporate_site/news+and+events/news/insights/bangladesh-garment-industry

[48] Government of Bangladesh. Bangladesh Economic Review 2019. Dhaka; 2019. Available: https://mof.portal.gov.bd/site/page/28ba57f5-59ff-4426-970a-bf014242179e/Bangladesh-Economic-Review

[49] United Nations. UN Comtrade database. In: Department of Economics and Social Affairs, Statistics Division, Trade Statistics, United Nations [Internet]. 2019 [cited 2 Sep 2019] pp. 1–4. Available: https://comtrade.un.org/data

[50] OECD. Coronavirus: The world economy at risk. OECD Interim Econ Assess. Paris; 2020. Available: https://www.oecd-ilibrary.org/docserver/7969896b-en.pdf?expires=1589469353&id=id&accname=guest&checksum=57E99A1484D296ACF13C1471FA3124DB

[51] UNDP. Assessment Report on Impact of COVID-19 Pandemic on Chinese Enterprises. United Nations Dev Program China, April 2020. New York; 2020. Available: https://www.cn.undp.org/content/china/en/home/library/crisis_prevention_and_recovery/assessment-report-on-impact-of-covid-19-pandemic-on-chinese-ente.html

[52] BGMEA. Impact of COVID-19 on Bangladesh RMG industry. In: Bangladesh Garment Manufacturers and Exporters Association (BGMEA) [Internet]. 2020 [cited 20 Aug 2020] pp. 1–2. Available: https://www.bgmea.com.bd/

[53] BBS. Labour Force Survey Bangladesh 2016–17. Dhaka; 2018. Available: http://203.112.218.65:8008/WebTestApplication/userfiles/Image/LatestReports/LFS_2016-17.pdf

[54] Government of Bangladesh. National Nutrition Policy 2015. Dhaka: Ministry of Health and Family Welfare, Government of Bangladesh; 2015. Available: http://etoolkits.dghs.gov.bd/sites/default/files/national_nutrition_policy-2015.pdf

[55] Raihan S. The political economy of the stimulus package for COVID-19 induced economic crisis in Bangladesh. SANEM Think Aloud. 2020;6: 1–2.

[56] Chowdhury SK, Rashid S, Khaled N Bin. Opinion on fighting on COVID-19 induced increasing poverty. The Daily Ittefaq, Dhaka, Bangladesh May 05, 2020. 5 May 2020. Available: https://www.ittefaq.com.bd/opinion/149542/

[57] BBS. Preliminary report on Households Income and Expenditure Survey 2016. Dhaka; 2017. Available: https://catalog.ihsn.org/index.php/catalog/7399/related-materials

[58] Mottaleb KA, Sonobe T. An Inquiry into the Rapid Growth of the Garment Industry in Bangladesh. Econ Dev Cult Change. 2011;60: 67–89. 10.1086/661218

[59] Vella F. Estimating Models with Sample Selection Bias: A Survey. J Hum Resour. 1998;33: 127 10.2307/146317

[60] Government of India. Annual Report: Periodic Labour Force Survey, 2017–18. Natl Stat Off. New Delhi; 2019. Available: http://mospi.nic.in/sites/default/files/publication_reports/Annual Report%2C PLFS 2017–18_31052019.pdf?download=1

[61] Vyas M. Job losses may have narrowed. In: Centre for Monitoring Indian Economy Pvt. Ltd. [Internet]. 2020 [cited 28 May 2020] pp. 44–46. Available: https://www.cmie.com/kommon/bin/sr.php?kall=warticle&dt=2020-05-2608:18:26&msec=533

[62] The Daily Prothom Alo. Governmet will provide monthly BDT 2000 to 2000000 hosueholds of Bangladesh. Stuff report. 7 May 2020. Available: https://www.prothomalo.com/economy/article/1655165/২০-লাখ-পরিবারকে-মাসে-২-হাজার-টাকা-করে-দেবে-সরকার

[63] Ahmed M. In May, 50,00000 households will get 20kg food support per month. The Daily Prothom Alo. 2 May 2020. Available: https://www.prothomalo.com/bangladesh/article/1654265/মে-মাসে-৫০-লাখ-পরিবার-২০-কেজি-করে-ত্রাণ-পাবে

[64] BBS. Report on Labour Force Survey (LFS) Bangladesh 2013. Dhaka; 2015. 10.1017/CBO9781107415324.004

