# Supplementary material for: COVID-19 induced economic loss and ensuring food security for vulnerable groups: Policy implications from Bangladesh
Source: PLoS One. 2020 Oct 16;15(10):e0240709. doi: 10.1371/journal.pone.0240709 (PMC7567397; doi:10.1371/journal.pone.0240709)
Supplement: S1 Table — (DOCX) [file pone.0240709.s001.docx]

S1 Table. District dummies included in explaining daily wage earnings (reported in Table 4) of the daily wage workers in the farm and nonfarm sectors (Bagerhat district is the base=0).

| Dependent variables | Daily wage rate (BDT) | |
| --- | --- | --- |
| Sampled sector | Farm sector | Nonfarm sector |
| Independent variables |  |  |
| Bandarban | 0.056** (0.02) | 0.050 (0.11) |
| Barguna | 0.080*** (0.02) | -0.054 (0.04) |
| Barishal | -0.075** (0.03) | -0.0047 (0.04) |
| Bhola | 0.076*** (0.02) | 0.18*** (0.03) |
| Bogura | -0.25*** (0.03) | -0.093** (0.04) |
| Brahmanbaria | 0.031 (0.04) | 0.014 (0.05) |
| Chandpur | -0.13 (0.08) | 0.096* (0.05) |
| Chattogram | 0.28*** (0.09) | 0.22*** (0.05) |
| Chuadanga | -0.20*** (0.07) | -0.23*** (0.08) |
| Cumilla | 0.11*** (0.03) | 0.29*** (0.04) |
| Cox’s Bazar | 0.15*** (0.02) | 0.19*** (0.05) |
| Dhaka |  | 0.26*** (0.05) |
| Dinajpur | -0.25*** (0.02) | -0.24*** (0.05) |
| Faridpur | -0.0089 (0.02) | 0.17*** (0.06) |
| Feni | 0.15*** (0.04) | 0.12*** (0.04) |
| Gaibandha | -0.35*** (0.03) | -0.18*** (0.04) |
| Gazipur | 0.068 (0.09) | 0.19*** (0.05) |
| Gopalganj | -0.28*** (0.04) | -0.024 (0.05) |
| Habiganj | -0.17*** (0.04) | -0.35*** (0.07) |
| Joypurhat | -0.22*** (0.03) | -0.28*** (0.05) |
| Jamalpur | 0.016 (0.05) | -0.069 (0.07) |
| Jashore | -0.26*** (0.03) | -0.14*** (0.04) |
| Jhalokati | -0.19*** (0.07) | -0.052 (0.04) |
| Jhenaidah | -0.34*** (0.03) | -0.23*** (0.04) |
| Khagrachari | -0.17*** (0.03) | -0.21*** (0.07) |
| Khulna | -0.074** (0.03) | -0.020 (0.04) |
| Kishoreganj | 0.079*** (0.03) | -0.096 (0.06) |
| Kurigram | -0.30*** (0.02) | -0.11** (0.04) |
| Khustia | -0.27*** (0.02) | -0.20*** (0.05) |
| Lakshmipur | 0.012 (0.04) | 0.079 (0.07) |
| Lalmonirhat | -0.33*** (0.02) | -0.35*** (0.11) |
| Madaripur | -0.17*** (0.03) | 0.22*** (0.04) |
| Magura | -0.15*** (0.02) | -0.20*** (0.07) |
| Manikganj | 0.083** (0.03) | 0.065 (0.05) |
| Meherpur | -0.47*** (0.02) | -0.29*** (0.04) |
| Maulvibazar | 0.28*** (0.08) | -0.31*** (0.06) |
| Munshiganj | 0.20*** (0.04) | 0.24*** (0.03) |
| Mymensingh | 0.35*** (0.06) | 0.41*** (0.05) |
| Naogaon | -0.32*** (0.03) | -0.45*** (0.06) |
| Narail | -0.14*** (0.02) | -0.015 (0.04) |
| Narayanganj | 0.052 (0.08) | 0.23*** (0.05) |
| Narsingdi | -0.084** (0.04) | 0.032 (0.05) |
| Natore | -0.23*** (0.03) | -0.25*** (0.05) |
| Chapai Nawabganj | -0.31*** (0.02) | -0.13** (0.06) |
| Netrokona | -0.12*** (0.04) | -0.17*** (0.06) |
| Nilphamari | -0.27*** (0.03) | -0.30*** (0.05) |
| Noakhali | 0.16** (0.07) | 0.064 (0.06) |
| Pabna | -0.022 (0.02) | -0.035 (0.03) |
| Panchagarh | -0.27*** (0.02) | -0.24*** (0.05) |
| Patuakhali | 0.0055 (0.02) | -0.081 (0.05) |
| Pirojpur | -0.012 (0.03) | 0.030 (0.05) |
| Rajshahi | -0.30*** (0.07) | -0.14** (0.06) |
| Rajbari | 0.049** (0.02) | 0.060 (0.04) |
| Rangamati | 0.022 (0.03) | 0.11 (0.07) |
| Rangpur | -0.35*** (0.02) | -0.38*** (0.04) |
| Shariatpur | -0.060* (0.03) | 0.13*** (0.04) |
| Satkhira | -0.40*** (0.03) | -0.29*** (0.04) |
| Sirajganj | -0.21*** (0.02) | -0.24*** (0.05) |
| Sherpur | 0.12*** (0.03) | 0.13** (0.05) |
| Sunamganj | -0.064** (0.03) | -0.057 (0.04) |
| Sylhet | -0.0045 (0.04) | 0.11** (0.05) |
| Tangail | -0.016 (0.04) | -0.039 (0.05) |
| Thakurgaon | -0.33*** (0.03) | -0.32*** (0.05) |

Notes: Values in parentheses are robust standard errors calculated applying bootstrap method replicating estimation 1000 times. ***, ** and * indicate the 1% level, 5% level and 10% level of significance, respectively.
