# Supplementary material for: COVID-19 induced economic loss and ensuring food security for vulnerable groups: Policy implications from Bangladesh
Source: PLoS One. 2020 Oct 16;15(10):e0240709. doi: 10.1371/journal.pone.0240709 (PMC7567397; doi:10.1371/journal.pone.0240709)
Supplement: S2 Table — Base district: Bagerhat = 0. (DOCX) [file pone.0240709.s002.docx]

S2 Table: District dummies included in explaining the daily household level total food expenditure (reported in Table 5). Base district: Bagerhat=0.

| Sector | Farm | Nonfarm |
| --- | --- | --- |
| Dependent variable | ln(Daily household level total food expenditure in BDT) | |
|  |  |  |
| Bandarban | -0.13*** (0.04) | -0.087 (0.07) |
| Barguna | 0.086* (0.05) | 0.048 (0.04) |
| Barishal | 0.071 (0.06) | -0.082* (0.04) |
| Bhola | 0.38*** (0.06) | 0.39*** (0.03) |
| Bogura | -0.15*** (0.06) | -0.21*** (0.04) |
| Brahmanbaria | 0.65*** (0.10) | 0.49*** (0.05) |
| Chandpur | 0.34*** (0.07) | 0.16*** (0.05) |
| Chattogram | 0.46** (0.19) | 0.29*** (0.03) |
| Chuadanga | 0.039 (0.04) | -0.0067 (0.05) |
| Cumilla | 0.34*** (0.06) | 0.27*** (0.04) |
| Cox’s Bazar | 0.56*** (0.05) | 0.54*** (0.03) |
| Dhaka |  | 0.094** (0.04) |
| Dinajpur | -0.24*** (0.04) | -0.19*** (0.04) |
| Faridpur | 0.37*** (0.05) | 0.32*** (0.04) |
| Feni | 0.28*** (0.07) | 0.27*** (0.04) |
| Gaibandha | -0.13** (0.06) | -0.093** (0.04) |
| Gazipur | 0.13 (0.08) | 0.13*** (0.04) |
| Gopalganj | 0.37*** (0.05) | 0.16*** (0.04) |
| Habiganj | 0.20*** (0.06) | 0.24*** (0.06) |
| Joypurhat | -0.12** (0.05) | -0.16*** (0.04) |
| Jamalpur | -0.14*** (0.05) | -0.15*** (0.04) |
| Jashore | 0.020 (0.05) | 0.044 (0.04) |
| Jhalokati | 0.071 (0.08) | 0.00012 (0.04) |
| Jhenaidah | 0.010 (0.05) | -0.022 (0.04) |
| Khagrachari | -0.045 (0.05) | -0.050 (0.05) |
| Khulna | 0.022 (0.06) | -0.18*** (0.03) |
| Kishoreganj | -0.0100 (0.05) | -0.022 (0.04) |
| Kurigram | -0.27*** (0.05) | -0.38*** (0.05) |
| Kushtia | 0.067 (0.05) | 0.11** (0.05) |
| Lakshmipur | 0.35*** (0.05) | 0.13** (0.06) |
| Lalmonirhat | 0.033 (0.04) | -0.0059 (0.05) |
| Madaripur | 0.54*** (0.05) | 0.38*** (0.04) |
| Magura | -0.017 (0.05) | 0.071 (0.04) |
| Manikganj | -0.095 (0.07) | -0.12** (0.05) |
| Meherpur | 0.010 (0.04) | -0.14*** (0.04) |
| Maulvibazar | 0.30*** (0.05) | 0.48*** (0.04) |
| Munshiganj | 0.34*** (0.06) | 0.29*** (0.04) |
| Mymensingh | -0.074 (0.07) | -0.11*** (0.04) |
| Naogaon | -0.13*** (0.04) | -0.057 (0.06) |
| Narail | 0.14*** (0.04) | 0.13*** (0.04) |
| Narayanganj | 0.27* (0.14) | 0.26*** (0.05) |
| Narsingdi | 0.27*** (0.07) | 0.42*** (0.04) |
| Natore | -0.041 (0.05) | 0.059 (0.07) |
| Chapai Nawabganj | -0.18*** (0.05) | -0.38*** (0.05) |
| Netrokona | 0.17*** (0.04) | 0.12** (0.05) |
| Nilphamari | 0.078* (0.05) | -0.14*** (0.04) |
| Noakhali | 0.29*** (0.06) | 0.33*** (0.05) |
| Pabna | 0.046 (0.05) | -0.033 (0.04) |
| Panchagarh | 0.13*** (0.05) | 0.011 (0.04) |
| Patuakhali | 0.24*** (0.07) | -0.076 (0.05) |
| Pirojpur | -0.039 (0.06) | -0.21*** (0.05) |
| Rajshahi | 0.26*** (0.07) | 0.016 (0.05) |
| Rajbari | 0.043 (0.05) | -0.14*** (0.04) |
| Rangamati | 0.25*** (0.04) | 0.27*** (0.05) |
| Rangpur | -0.15*** (0.05) | -0.19*** (0.04) |
| Shariatpur | 0.33*** (0.05) | 0.26*** (0.04) |
| Satkhira | 0.052 (0.04) | -0.051 (0.03) |
| Sirajganj | 0.11** (0.05) | 0.064 (0.04) |
| Sherpur | 0.15*** (0.05) | 0.089** (0.04) |
| Sunamganj | 0.24*** (0.04) | 0.29*** (0.04) |
| Sylhet | 0.41*** (0.08) | 0.35*** (0.05) |
| Tangail | 0.048 (0.07) | 0.082 (0.06) |
| Thakurgaon | 0.049 (0.05) | 0.097** (0.04) |

Notes: Values in parentheses are robust standard errors calculated applying bootstrap method replicating estimation 1000 times. ***, ** and * indicate the 1% level, 5% level and 10% level of significance, respectively.
