# Supplementary material for: COVID-19 induced economic loss and ensuring food security for vulnerable groups: Policy implications from Bangladesh
Source: PLoS One. 2020 Oct 16;15(10):e0240709. doi: 10.1371/journal.pone.0240709 (PMC7567397; doi:10.1371/journal.pone.0240709)
Supplement: S3 Table — Base district: Bagerhat. (DOCX) [file pone.0240709.s003.docx]

S3 Table: District dummies included in explaining the occupation choice (reported in Table 7). Base district : Bagerhat

|  | Base: Workers based on weekly or monthly basis payment system(=0) | |
| --- | --- | --- |
| Dependent variable | Daily basis worker | |
|  | Farm sector  (yes=1) | Nonfarm sector (yes=2) |
| District dummies |  |  |
| Base district: Bagerhat(=0) |  |  |
| Bandarban | -0.42** (0.17) | -2.38*** (0.25) |
| Barguna | -0.87*** (0.20) | -0.62*** (0.15) |
| Barishal | -0.63*** (0.19) | -0.48*** (0.16) |
| Bhola | -0.99*** (0.19) | -0.17 (0.14) |
| Bogura | -0.75*** (0.18) | -0.56*** (0.15) |
| Brahmanbaria | -1.20*** (0.19) | -1.12*** (0.16) |
| Chandpur | -0.63*** (0.19) | -0.65*** (0.17) |
| Chattogram | -2.20*** (0.52) | -0.40** (0.20) |
| Chuadanga | -0.072 (0.16) | -1.40*** (0.17) |
| Cumilla | -0.76*** (0.20) | -0.64*** (0.16) |
| Cox’s Bazar | 0.24 (0.17) | -0.29** (0.15) |
| Dhaka | -25.5*** (0.15) | -1.20*** (0.17) |
| Dinajpur | 0.12 (0.16) | -0.66*** (0.15) |
| Faridpur | 0.11 (0.17) | -0.57*** (0.15) |
| Feni | -1.12*** (0.24) | -0.24 (0.17) |
| Gaibandha | 0.20 (0.17) | -0.12 (0.15) |
| Gazipur | -2.32*** (0.39) | -1.40*** (0.19) |
| Gopalganj | 0.24 (0.17) | -0.17 (0.15) |
| Habiganj | -0.86*** (0.18) | -1.24*** (0.16) |
| Joypurhat | -0.30* (0.17) | -1.04*** (0.15) |
| Jamalpur | -0.42** (0.17) | -1.27*** (0.16) |
| Jashore | -0.14 (0.18) | -0.74*** (0.15) |
| Jhalokati | -2.21*** (0.32) | -0.56*** (0.16) |
| Jhenaidah | -0.52** (0.21) | -0.78*** (0.17) |
| Khagrachari | -0.40** (0.19) | -1.74*** (0.21) |
| Khulna | -0.71*** (0.23) | -0.41*** (0.15) |
| Kishoreganj | -0.74*** (0.17) | -1.12*** (0.16) |
| Kurigram | 0.20 (0.17) | -0.49*** (0.15) |
| Kushtia | -0.34** (0.17) | -1.07*** (0.15) |
| Lakshmipur | -0.15 (0.17) | -0.67*** (0.17) |
| Lalmonirhat | 0.13 (0.16) | -1.41*** (0.16) |
| Madaripur | -0.72*** (0.18) | -0.72*** (0.15) |
| Magura | -0.39** (0.18) | -1.98*** (0.19) |
| Manikganj | -1.68*** (0.21) | -1.23*** (0.16) |
| Meherpur | 0.38** (0.17) | -0.78*** (0.16) |
| Maulvibazar | 0.23 (0.17) | -0.79*** (0.16) |
| Munshiganj | -0.63*** (0.21) | -0.29* (0.16) |
| Mymensingh | -0.84*** (0.19) | -0.84*** (0.16) |
| Naogaon | 0.42*** (0.16) | -1.05*** (0.16) |
| Narail | 0.38** (0.17) | -0.44*** (0.15) |
| Narayanganj | -2.50*** (0.46) | -1.23*** (0.18) |
| Narsingdi | -1.35*** (0.23) | -0.81*** (0.17) |
| Natore | 0.00081 (0.17) | -1.26*** (0.16) |
| Chapai Nawabganj | -0.52*** (0.17) | -0.86*** (0.15) |
| Netrokona | 0.24 (0.17) | -1.43*** (0.18) |
| Nilphamari | 0.72*** (0.17) | -0.52*** (0.16) |
| Noakhali | -0.12 (0.18) | -0.60*** (0.17) |
| Pabna | -0.28 (0.17) | -0.80*** (0.15) |
| Panchagarh | -0.0041 (0.16) | -1.22*** (0.16) |
| Patuakhali | -1.46*** (0.19) | -1.65*** (0.16) |
| Pirojpur | -1.04*** (0.19) | -0.78*** (0.15) |
| Rajshahi | 0.33 (0.23) | -0.91*** (0.24) |
| Rajbari | -0.19 (0.19) | -0.50*** (0.17) |
| Rangamati | -0.92*** (0.18) | -1.86*** (0.33) |
| Rangpur | -0.42** (0.17) | -0.66*** (0.15) |
| Shariatpur | -0.65*** (0.19) | -0.92*** (0.17) |
| Satkhira | 0.44*** (0.16) | -0.082 (0.14) |
| Sirajganj | -0.40** (0.17) | -0.71*** (0.15) |
| Sherpur | -0.23 (0.17) | -0.72*** (0.15) |
| Sunamganj | 0.076 (0.17) | -0.58*** (0.15) |
| Sylhet | -1.08*** (0.21) | -0.063 (0.15) |
| Tangail | -1.32*** (0.22) | -1.11*** (0.18) |
| Thakurgaon | -0.29 (0.18) | -1.07*** (0.16) |

Notes: Values in parentheses are robust standard errors calculated applying bootstrap method replicating estimation 1000 times. ***, ** and * indicate the 1% level, 5% level and 10% level of significance, respectively.
